# Evaluation of multimodal large language models for psoriasis diagnosis, severity grading, and treatment recommendations from clinical photographs: ChatGPT shows superior performance compared to other large language models

**DOI:** 10.3389/fmed.2026.1791488

**Published:** 2026-05-13

**Authors:** Mehdi Boostani, Paolo Gisondi, Francesco Bellinato, Tara Kiss, Christos C. Zouboulis, Drew Kuraitis, Noah Goldfarb, Nóra Nádudvari, Banu Farabi, Balazs Hodosi, Péter Holló, Norbert M. Wikonkal, Kende Lőrincz, András Banvölgyi, Gyorgy Paragh, Norbert Kiss

**Affiliations:** 1 Department of Dermatology, Roswell Park Comprehensive Cancer Center, Buffalo, NY, United States; 2 Department of Medicine, Section of Dermatology, University of Verona, Verona, Italy; 3 Department of Dermatology, Venereology and Dermatooncology, Semmelweis University, Budapest, Hungary; 4 Brandenburg Medical School Theodor Fontane and Faculty of Health Sciences Brandenburg, Neuruppin, Germany; 5 Departments of Medicine and Dermatology, University of Minnesota, Minneapolis, MN, United States; 6 Departments of Medicine and Dermatology, Minneapolis VA Health Care System, Minneapolis, MN, United States; 7 Dermatology Department, Icahn School of Medicine, Mount Sinai Hospital, New York, NY, United States; 8 Kings College Hospital London, Gulf Medical University, Dubai, United Arab Emirates; 9 Central Hospital of Northern Pest–Military Hospital, Budapest, Hungary

**Keywords:** AI, artificial intelligence, ChatGPT, Claude, diagnosis, Gemini, large language model, psoriasis

## Abstract

**Introduction:**

Psoriasis is common, but diagnosis and early severity assessment can be delayed because of variable presentation and overlap with mimicking dermatoses. Multimodal large language models (LLMs) may assist image-based triage.

**Objective:**

To evaluate web-based multimodal LLMs for psoriasis identification, Physician Global Assessment (PGA) scoring, and treatment recommendation quality from clinical photographs.

**Methods:**

We retrospectively analyzed 303 standardized photographs from 160 patients (Semmelweis University, May 2022–January 2025), including 163 psoriasis lesions and 140 mimickers. Reference diagnosis and PGA were assigned by two dermatologists with third-expert adjudication, treatment outputs were rated for appropriateness. ChatGPT-5, ChatGPT-4o, Gemini 2.5 Flash, and Claude Sonnet 4.5 received identical prompts for diagnosis and, when psoriasis was predicted, PGA and treatment; sessions were periodically reset.

**Results:**

Diagnostic accuracy was highest for ChatGPT-5 (93.1%) and ChatGPT-4o (90.1%), followed by Claude (83.6%) and Gemini (61.5%). Among correctly identified psoriasis cases, PGA accuracy was 93.3% (ChatGPT-5), 92.9% (ChatGPT-4o), 82.7% (Gemini), and 78.1% (Claude). Appropriate treatment recommendations were most frequent for ChatGPT-5 (83.7%) and ChatGPT-4o (82.1%), then Claude (75.0%) and Gemini (53.2%); test–retest agreement favored the OpenAI models.

**Conclusion:**

Under standardized conditions, general-purpose multimodal LLMs, especially ChatGPT-5 and ChatGPT-4o, showed strong performance for psoriasis recognition and reasonable support for PGA scoring and treatment suggestions, supporting potential use by primary care physicians when dermatology specialist access is limited.

## 1 Introduction

Psoriasis is a chronic, immune-mediated inflammatory skin disease that affects between 0.91 and 8.5% of adults worldwide, imposing substantial individual and societal burden (1). Despite published diagnostic criteria, recognition in primary care remains challenging due to variability in presentation, overlapping morphologies with common mimickers (e.g., eczema, tinea) (2–5), and under-recognition, particularly on darker skin tones and in special sites, can delay treatment initiation and prolong morbidity (1, 6, 7). Such delays are associated with persistent cutaneous symptoms, impaired functioning, and psychological comorbidities including anxiety, depression, and stigma (8–11). Early, accurate diagnosis is therefore essential to expedite evidence-based therapy and mitigate downstream consequences.

Standardized severity assessment further supports timely care. The Physician Global Assessment (PGA) is widely used in trials and routine practice to rate overall and lesion-level disease severity and to track treatment response, providing a pragmatic anchor for clinical decision-making (12).

Large language models (LLMs) are deep neural networks trained on massive text corpora that learn statistical patterns of language to understand, generate, and transform human-like text. When equipped with vision inputs (multimodal LLMs), these systems can jointly process images and text, enabling image-informed reasoning and triage suggestions relevant to clinical workflows (13–16). Given their broad availability and rapid iteration, evaluating how off-the-shelf, web-based LLMs perform on common dermatologic tasks is timely. Therefore, this study aimed to assess the diagnostic accuracy, PGA scoring, and treatment recommendation performance of such LLMs using clinical photographs of psoriasis.

## 2 Methods

### 2.1 Study design and data source

We conducted a retrospective evaluation of clinical photographs from patients with untreated psoriasis diagnosed at Semmelweis University between May 2022 and January 2025. Eligibility required a clinical or histopathologic diagnosis of psoriasis prior to imaging. In addition to confirmed psoriasis cases, we included a psoriasis-mimicker cohort to enable head-to-head discrimination. This cohort consisted of clinical photographs from patients with non-psoriatic conditions that commonly resemble psoriasis in routine practice, captured under identical photography conditions. All images were captured by a clinical photographer using a digital single-lens reflex (DSLR) camera under standardized conditions, including controlled lighting and fixed image-to-lesion distance of 15 to 30 centimeters. At the time of imaging, patients routinely provided written informed consent for clinical photography and for the use of their de-identified images in research, including AI-based evaluation and training. In accordance with institutional policy for retrospective analyses of anonymized, routinely collected clinical data, this study was considered exempt from additional institutional review board/ethics committee approval. The ethical justification for the present study was based on the secondary analysis of previously collected, de-identified clinical images obtained with consent, minimal risk to participants, and the non-interventional nature of the investigation. Consistent with responsible-AI principles in medicine, the evaluated models were assessed only as experimental clinician-support tools and not as autonomous systems for unsupervised diagnosis or treatment decision-making.

### 2.2 Models and inference protocol

We assessed four web-based multimodal, non-task-trained LLMs: ChatGPT-4o and ChatGPT-5 (OpenAI, San Francisco, CA, USA), Gemini 2.5 Flash (Google, Mountain View, CA, USA), and Claude Sonnet 4.5 (Anthropic, San Francisco, CA, USA). Each clinical photograph was evaluated individually and under the same standardized prompting for all models.

For each image, the model was first provided with the clinical photograph and the following initial prompt: “Can you guess the most likely diagnosis?” No additional clinical history, demographic information, lesion location, or differential diagnosis list was provided.

If, and only if, the model identified the lesion as psoriasis, a second prompt was issued in the same session: “For this case of psoriasis, can you estimate the most appropriate PGA score and treatment recommendation?” If the model did not identify psoriasis in response to the first prompt, no follow-up PGA or treatment prompt was given for that image.

All images were tested using the same prompt wording and the same prompt order across all models. To minimize carry-over effects and memory-related bias during sequential testing, active model sessions were reset and memory was cleared after every five image evaluations. No model-specific fine-tuning, system instruction customization, or task-specific training was applied.

### 2.3 Reference standard, grading, and endpoints

For each clinical photograph, two board-certified dermatologists (AB and KL) with more than 5 years’ experience in psoriasis management independently reviewed the image while blinded to model outputs. First, they determined whether the lesion represented psoriasis or a non-psoriatic condition. In cases of discordant assessments, a third expert dermatologist (NK) adjudicated the final reference diagnosis.

For contextual reference, all images were also independently evaluated by the same three blinded dermatologists for psoriasis identification, using the onsite dermatologist’s diagnosis as the reference standard. In this dataset, all three dermatologists achieved 100% diagnostic accuracy.

**Figure 1:**
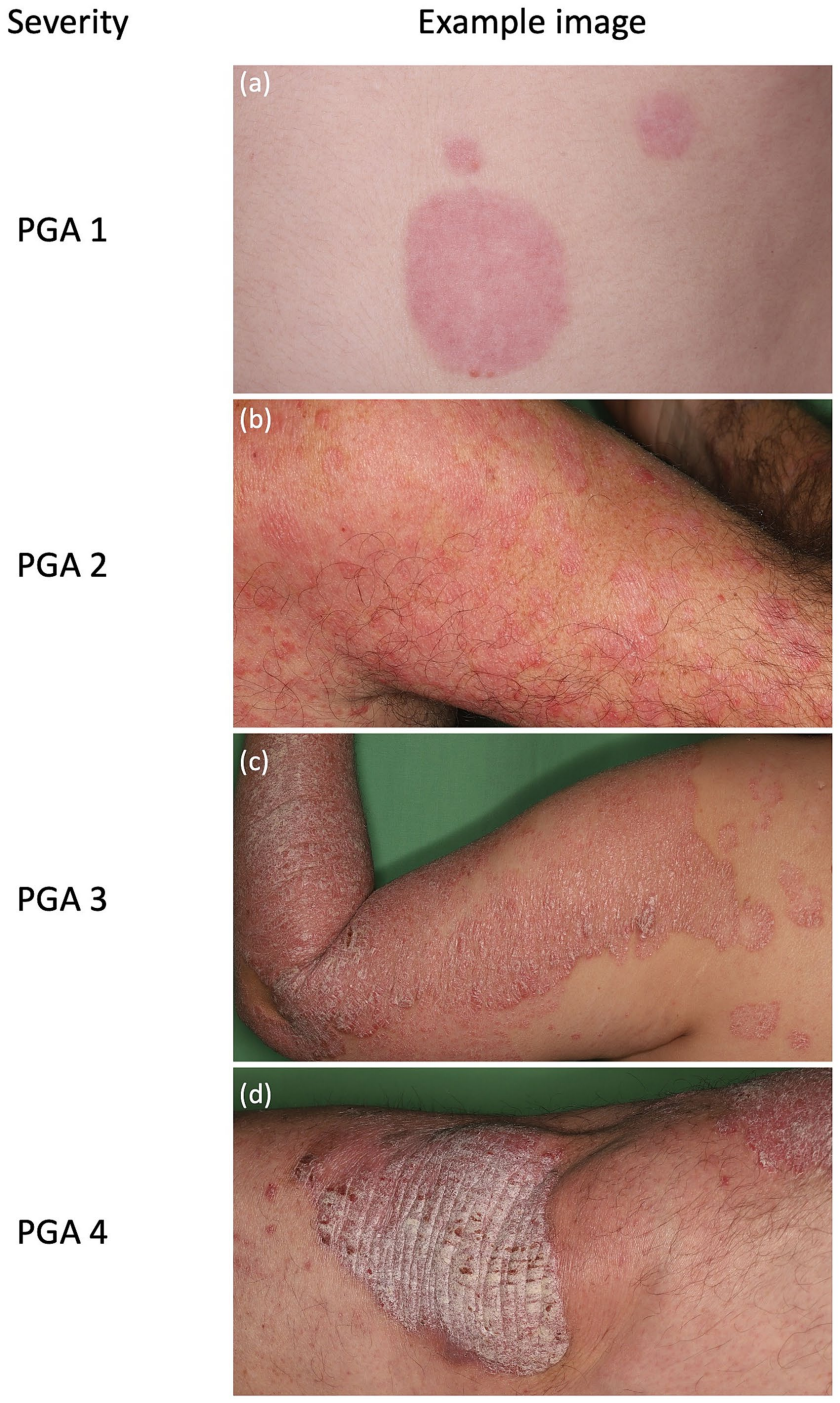
Representative clinical photographs illustrating psoriasis Physician Global Assessment severity categories. Standardized clinical images of plaque psoriasis exemplifying the four Physician Global Assessment (PGA) severity categories used in this study. **(a)** PGA 1: Faint, well-demarcated erythematous patch with minimal scaling; **(b)** PGA 2: Mild disease with multiple thin erythematous plaques and fine scale on the extremity; **(c)** PGA 3: Moderate psoriasis with confluent plaques showing increased thickness and scaling; **(d)** PGA 4: Severe psoriasis with very thick, hyperkeratotic plaques, and fissuring.

For images classified as psoriasis according to this reference diagnosis, the same two dermatologists independently assigned a PGA score (I–IV) based on erythema, induration, and scaling. (Figure 1) Disagreements in PGA grading were resolved by the third dermatologist, whose decision defined the reference PGA score for that image.

For these confirmed psoriasis cases, model-generated treatment recommendations were independently evaluated by the same two dermatologists and categorized as appropriate, partially appropriate, or inappropriate according to current clinical guidelines and clinical suitability for the lesion severity shown. Recommendations were considered appropriate if they were guideline-consistent and well matched to severity, partially appropriate if they were generally reasonable but incomplete, overly broad, or not optimally tailored, and inappropriate if they were inconsistent with accepted psoriasis management or clearly mismatched to the case. Any disagreement was resolved by the third dermatologist. For quantitative analysis, treatment recommendation performance was defined as the proportion of correctly identified psoriasis cases receiving an appropriate rating.

Primary endpoints were: (1) diagnostic accuracy for psoriasis; (2) PGA scoring accuracy among correctly diagnosed cases; and (3) appropriateness of treatment recommendations. A 30-case subset was used to assess test–retest agreement for diagnosis, PGA, and treatment outputs.

### 2.4 Statistical analysis and sample size

A priori sample-size calculations for diagnostic performance were performed for estimating the proportion of psoriasis cases correctly identified (diagnostic sensitivity), based on confidence-interval methods for binomial proportions. For psoriasis recognition, we assumed an expected sensitivity of 90%, an absolute precision of ±10% around this estimate, and a 95% confidence level. We additionally allowed for up to 10% unusable images (e.g., poor image quality or missing reference grading). Under these assumptions, the required sample size for estimating diagnostic sensitivity was 139 evaluable images, corresponding to a target of 155 images after accounting for unusable cases.

Diagnostic performance for psoriasis identification was summarized using sensitivity, specificity, positive predictive value (PPV), negative predictive value (NPV), and overall accuracy. These metrics were calculated by comparing each model’s categorical output against the reference diagnosis for each clinical photograph. For all primary performance measures, 95% confidence intervals were calculated using the Wilson method.

PGA scoring performance was defined as the proportion of correctly identified psoriasis cases for which the model-assigned PGA matched the reference PGA score. Treatment recommendation performance was defined as the proportion of correctly identified psoriasis cases for which the recommendation was rated as appropriate. Corresponding 95% confidence intervals were also estimated using binomial methods.

Test–retest agreement was assessed in a 30-case subset by repeating the full prompting workflow in reset sessions and calculating percentage agreement for diagnosis, PGA, and treatment recommendation category across the two runs. Composite repeatability was defined as the arithmetic mean of agreement across these three tasks.

## 3 Results

### 3.1 Cohort and image set

A total of 303 clinical photographs, 163 psoriasis lesions plus 140 psoriasis mimicker lesions, from 160 patients, 80 psoriasis patients plus 80 patients with conditions that can mimic psoriasis, were analyzed. In the psoriasis cohort, the mean number of lesions per patient was 2.04 ± 1.43. In the psoriasis mimicker cohort, the mean number of lesions per patient was 1.75 ± 0.9. Detailed demographics of the psoriasis patients and lesions are provided in Table 1, and detailed demographics of the psoriasis mimicker patients and lesions are provided in Table 2.

**Table 1:** Patient and lesion characteristics in the psoriasis cohort.

Category	Total (*n* = 80)
Patient characteristics	
Age (years)	
Mean	51.2 ± 16.1
Median	50
Range	20–82
Sex	
Male	60% (48)
Female	40% (32)
Fitzpatrick skin type	
Type II	85% (68)
Type III	15% (12)
Lesion characteristics	
Lesion distribution	
Extremity	62.6% (102)
Trunk	30% (49)
Head and neck	7.4% (12)
PGA score	
I	4.3% (7)
II	21.5% (35)
III	45.4% (74)
IV	28.8% (47)
Subtypes	
Plaque	89% (145)
Erythrodermic	5.5% (9)
Guttate	3.1% (5)
Palmoplantar	1.8% (3)
Nail	0.6% (1)

Descriptive demographic characteristics of the enrolled patients in the psoriasis cohort and descriptive characteristics of the psoriasis lesions included in the study. Continuous variables are presented as mean ± standard deviation, median, and range. Categorical variables are presented as percentage (count). PGA, physician global assessment.

**Table 2:** Demographic and lesion characteristics of the psoriasis mimicker cohort.

Category	Total (*n* = 80)
Patient characteristics	
Age (years)	
Mean	49.8 ± 15.7
Median	50
Range	19–81
Sex	
Male	55% (44)
Female	45% (36)
Fitzpatrick skin type	
Type I	5% (4)
Type II	62.5% (50)
Type III	25% (20)
Type IV	7.5% (6)
Lesion characteristics	
Lesion distribution	
Extremity	60% (84)
Trunk	32.9% (46)
Head and neck	7.1% (10)
Diagnosis	
Atopic dermatitis	20% (28)
Contact dermatitis	20% (28)
Dermatitis herpetiformis	11.4% (16)
Pityriasis rubra pilaris	10% (14)
Nummular eczema	10% (14)
Tinea	10% (14)
Seborrheic dermatitis	10% (14)
Mycosis fungoides	5% (7)
Lichen planus	3.6% (5)

Descriptive demographic characteristics of the patients in the psoriasis mimicker cohort and descriptive characteristics of the photographed lesions included in the study. Continuous variables are presented as mean ± standard deviation, median, and range. Categorical variables are presented as percentage (count). Lesion distribution indicates the anatomic location of the photographed lesion, and diagnosis refers to the final clinical diagnosis assigned to each mimicker case.

### 3.2 Psoriasis identification

ChatGPT-5 achieved the highest diagnostic accuracy at 93.1% (95% CI: 89.7–95.4), with a sensitivity of 93.3% (95% CI: 88.4–96.2) and specificity of 92.9% (95% CI: 87.4–96.1). This was followed by ChatGPT-4o, with an accuracy of 90.1% (95% CI: 86.3–93.0), sensitivity of 89.0% (95% CI: 83.3–92.9), and specificity of 91.4% (95% CI: 85.6–95.0). Claude Sonnet 4.5 demonstrated an accuracy of 83.6% (95% CI: 79.0–87.3), sensitivity of 78.0% (95% CI: 71.1–83.7), and specificity of 90.0% (95% CI, 83.9–93.9), whereas Gemini 2.5 Flash showed an accuracy of 61.5% (95% CI, 55.9–66.8) with markedly lower sensitivity (37.8% [95% CI, 30.7–45.4]) despite a relatively preserved specificity (89.3% [95% CI, 83.1–93.4]). Additional diagnostic performance metrics are summarized in Table 3. For contextual reference, all three blinded dermatologists achieved 100% diagnostic accuracy for psoriasis identification relative to the onsite dermatologist’s diagnosis.

**Table 3:** Diagnostic performance of large language models for psoriasis identification.

Model	Sensitivity (95% CI)	Specificity (95% CI)	PPV (95% CI)	NPV (95% CI)	Accuracy (95% CI)
ChatGPT-4o	89.0% (83.3–92.9%)	91.4% (85.6–95.0%)	92.4% (87.2–95.6%)	87.7% (81.4–92.1%)	90.1% (86.3–93.0%)
ChatGPT-5	93.3% (88.4–96.2%)	92.9% (87.4–96.1%)	93.9% (89.1–96.6%)	92.2% (86.6–95.6%)	93.1% (89.7–95.4%)
Claude sonnet 4.5	78.0% (71.1–83.7%)	90.0% (83.9–93.9%)	90.1% (84.1–94.0%)	77.8% (70.8–83.5%)	83.6% (79.0–87.3%)
Gemini 2.5 flash	37.8% (30.7–45.4%)	89.3% (83.1–93.4%)	80.5% (70.3–87.8%)	55.1% (48.6–61.4%)	61.5% (55.9–66.8%)

Diagnostic performance metrics are reported for each model in distinguishing psoriasis from psoriasis mimickers using clinical photographs. Metrics include sensitivity, specificity, positive predictive value, negative predictive value, and overall accuracy. Values are presented as percentage (95% confidence interval). Confidence intervals were calculated using the Wilson method. CI, confidence interval, NPV, negative predictive value, PPV, positive predictive value.

### 3.3 PGA scoring accuracy

Among the correctly identified psoriasis cases, PGA accuracy was highest for ChatGPT-5 at 93.3% (95% CI: 91–95), followed by ChatGPT-4o at 92.9% (95% CI: 90.5–94.9), Gemini 2.5 Flash at 82.7% (95% CI: 77.4–87.2), and Claude Sonnet 4.5% at 78.1% (95% CI: 74.3–81.6).

### 3.4 Treatment recommendation appropriateness

Among the correctly identified psoriasis cases, appropriateness of treatment recommendations was highest for ChatGPT-5 at 83.7% (95% CI: 77–88.7), followed by ChatGPT-4o at 82.1% (95% CI: 75–87.5), Claude Sonnet 4.5 at 75.0% (95% CI: 66.8–81.7), and Gemini 2.5 Flash at 53.2% (95% CI: 41–65.1).

### 3.5 Test–retest agreement

In a 30-case subset, test–retest agreement showed composite repeatability was highest for ChatGPT-5 (96.7%), followed closely by ChatGPT-4o (95.0%), and was lower for Claude Sonnet 4.5 (90.8%) and Gemini 2.5 Flash (88.3%), indicating a clear stability gradient favoring the OpenAI models (Table 4).

**Table 4:** Test–retest agreement and composite repeatability of model outputs on a 30-image subset.

Model	Diagnosis (%)	PGA (%)	Treatment (%)	Composite repeatability (%)
ChatGPT-5	100	95	95	96.7
ChatGPT-4o	95	95	95	95
Claude Sonnet 4.5	92.5	90	90	90.8
Gemini 2.5 Flash	87.5	87.5	90	88.3

Agreement (%) across two independent runs for three tasks, diagnosis label, PGA grade, and treatment category, with sessions reset and memory cleared between runs. Composite repeatability is the arithmetic mean of the three task agreements; higher values indicate greater output stability.

## 4 Discussion

**Figure 2:**
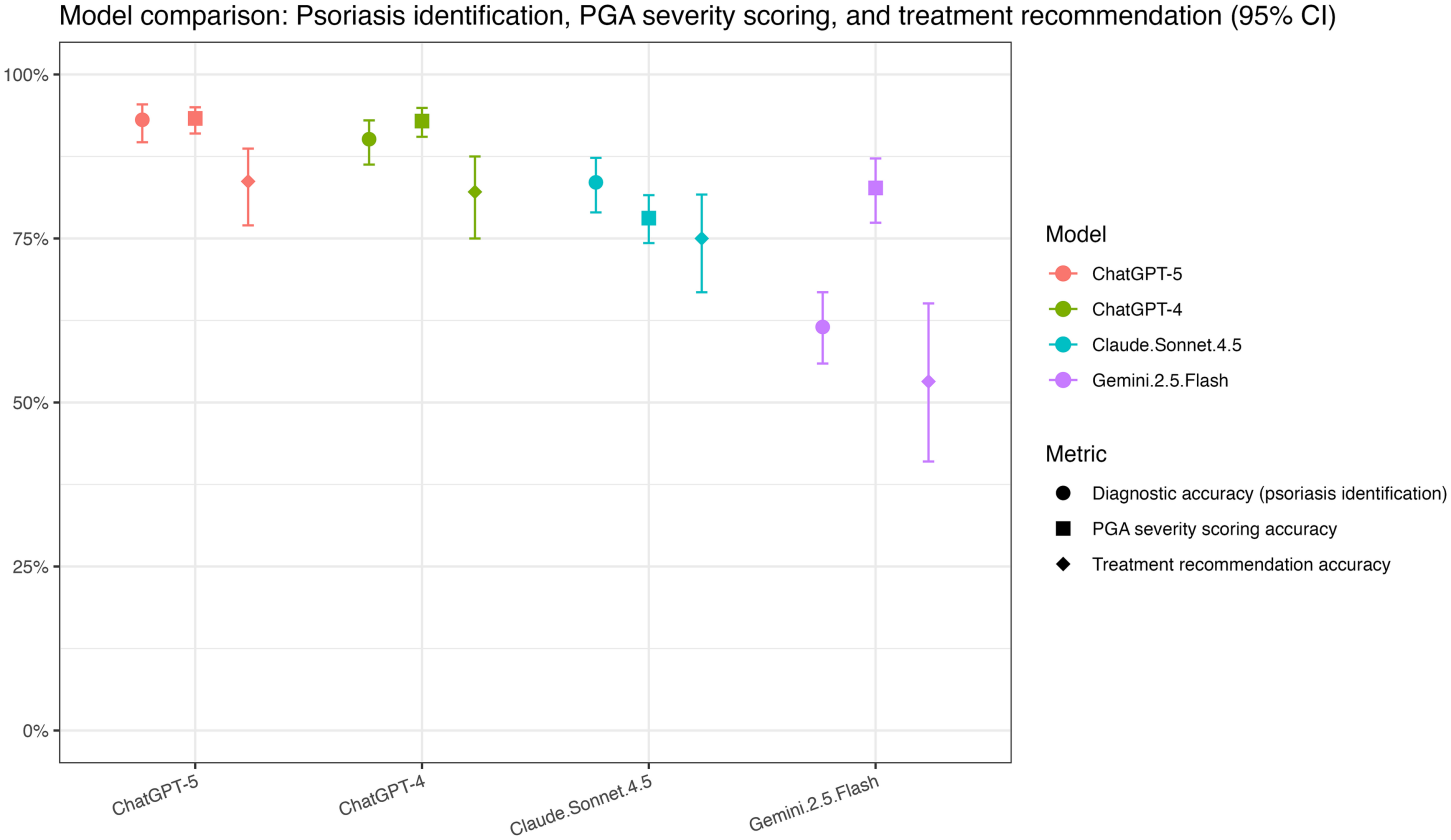
Comparison of large language model performance for psoriasis identification, PGA severity scoring, and treatment recommendation accuracy. Point estimates and 95% confidence intervals for three performance outcomes across models (ChatGPT-5, ChatGPT-4o, Claude Sonnet 4.5, and Gemini 2.5 Flash). Diagnostic accuracy (psoriasis identification) reflects overall accuracy for distinguishing psoriasis from psoriasis mimickers. PGA severity scoring accuracy and treatment recommendation accuracy are calculated among correctly identified psoriasis cases. Models are color-coded, and metrics are distinguished by marker shape. Error bars indicate 95% confidence intervals.

Our findings underscore the emerging potential of multimodal LLMs for dermatologic support. Overall, while ChatGPT-5 demonstrated the highest performance across diagnostic accuracy, PGA scoring, and treatment recommendation (Figure 2), its advantage over ChatGPT-4o was modest, reflecting broadly similar capabilities between the two models.

Importantly, dermatologist performance in this dataset was uniformly perfect, with all three blinded dermatologists achieving 100% diagnostic accuracy for psoriasis identification relative to the onsite dermatologist’s diagnosis. This finding indicates that, although the best-performing LLMs showed promising results, they still did not reach dermatologist-level performance in the present study.

Previous convolutional neural network (CNN)-based psoriasis classifiers have reported high performance on curated datasets. Zhao et al. (17) trained a two-stage CNN on clinical images and achieved a 98% specificity, 92% sensitivity, and 96% accuracy. Yang et al. (18) used dermoscopic images to train an EfficientNet-B4 CNN, reporting 92.9% sensitivity and 95.2% specificity for psoriasis. Yu et al. (19) developed a dermoscopic CNN for scalp psoriasis vs. seborrheic dermatitis with 96.1% sensitivity, 88.2% specificity.

In parallel, task-trained AI systems have shown strong performance in psoriasis severity assessment. Huang et al. developed a deep learning model trained on more than 14,000 images to estimate PASI scores, achieving a mean absolute error of 2.05 and outperforming 43 experienced dermatologists by 33.2% in overall PASI scoring accuracy. The model was successfully deployed in a real-world app (SkinTeller), where its utility was confirmed across 18 hospitals and over 1,400 patients, demonstrating how specialized AI systems can deliver high accuracy and scalability in clinical practice (20). Complementing these reports, Okamoto et al. (21) introduced a ‘Single-Shot PASI’ (SS-PASI) approach that estimates erythema, induration, scaling, area, and a composite severity score from a single trunk photograph using a fine-tuned InceptionV3 model. In a held-out test set, AI scores closely matched an expert’s labels and, notably, AI assistance reduced scoring variance and improved inter-rater concordance among 13 dermatologists and 9 medical students. Although the dataset was relatively small (705 trunk images; 10-image test set) and limited to standardized trunk photographs, the study illustrates how task-specific deep learning can streamline psoriasis severity scoring. It also demonstrates how such models can harmonize assessments in practice, paralleling, but remaining distinct from, our exploratory evaluation of general-purpose LLMs (21).

Beyond psoriasis, recent work has also demonstrated promising LLM performance in melanoma diagnosis. Recent analysis on ChatGPT-4o and Gemini 2.0 Flash on clinical and dermoscopic images to distinguish melanoma from nevi, report sensitivities up to 96.5% and specificities up to 98.8% (22). Moreover, multimodal LLMs have also been evaluated in other inflammatory dermatoses. A 2025 study assessed ChatGPT-4o, Gemini 2.0 Flash, and Claude Sonnet 3.7 on clinical images of hidradenitis suppurativa, demonstrating diagnostic accuracies up to 87.3%, staging accuracies approaching 82.6%, and treatment recommendation appropriateness nearing 88.7% for ChatGPT-4o (14). These findings parallel our results in psoriasis and melanoma, further underscoring the rapid evolution of LLM capabilities across a spectrum of dermatologic tasks.

Importantly, the potential risks associated with incorrect or overconfident LLM outputs must be carefully considered. Erroneous diagnostic or treatment suggestions could lead to inappropriate reassurance, delayed specialist referral, or mismanagement, especially in settings with limited dermatologic expertise. These issues raise important medicolegal considerations, including questions of responsibility when AI-assisted recommendations influence clinical decision-making. As such, robust safeguards, human oversight, and clear accountability frameworks are critical for safe deployment. While ChatGPT-5 should not be used independently by patients, current evidence supports its integration into primary care as a clinician-supervised decision aid. Its role is not to make a diagnosis but to assist with psoriasis triaging and help prevent delays in referral to specialists.

From an ethical perspective, the findings should be interpreted within established principles for the responsible use of AI in healthcare, including transparency, human oversight, protection of patient privacy, attention to fairness and bias, and validation before clinical deployment. In this study, the models were evaluated in a retrospective research setting using de-identified images, and all outputs were assessed against dermatologist-derived reference standards rather than used for patient care. Nevertheless, because LLM outputs may be inaccurate, overconfident, or inconsistently calibrated, any future clinical use would require careful governance, prospective evaluation, external validation, and clear clinician accountability. These considerations are particularly important in dermatology, where bias related to skin tone representation and image acquisition conditions may affect both performance and equity.

### 4.1 Limitations

Limitations of this study include a single-center, standardized image set with only of Fitzpatrick II–III skin types, and categorical grading of treatment appropriateness. Furthermore, the image set predominantly comprised plaque-type psoriasis, whereas other clinical subtypes were only sparsely represented. Importantly, we did not perform a formal same-dataset comparison with established CNN-based classifiers under the same conditions. Accordingly, interpretation of the relative value of LLMs versus benchmark AI models remains limited and should be considered an important limitation. Furthermore, web-based LLMs may evolve rapidly, necessitating periodic reevaluation and human oversight. Additionally, because no standardized, widely adopted diagnostic algorithms for psoriasis exist, benchmarking typically relies on dermatologist assessments or task-specific CNN-based models, which were not included in this study. An additional limitation is the potential for bias related to both skin tone representation and image acquisition conditions. Our dataset predominantly included patients with Fitzpatrick skin types II–III, with limited representation of darker skin tones, and this may reduce the generalizability of our findings across more diverse patient populations. Because psoriasis and its mimickers may present differently across skin tones, model performance observed in this study may not be maintained in underrepresented groups. Lastly, all photographs were obtained under standardized conditions using a DSLR camera, controlled lighting, and a fixed image-to-lesion distance. Although this approach improved internal consistency, it may also have introduced spectrum and acquisition bias by creating a more uniform image set than is typically encountered in routine practice. Furthermore, because this was a single-center study and no independent external validation cohort was available, the extent to which these findings generalize to other clinical settings remains uncertain. Real-world images obtained in primary care or by patients may vary substantially in lighting, focus, framing, background, resolution, and color balance, all of which could affect model performance. Therefore, the results of the present study should be interpreted as reflecting performance under controlled imaging conditions rather than fully representative real-world use, and confirmation in external, multi-center datasets will be necessary.

Future work should expand to multi-center datasets, include more diverse skin tones, and incorporate community-captured images. It should also include true differential diagnosis against common mimickers and direct head-to-head comparison of LLMs with task-specific CNN models under identical conditions, alongside calibration and uncertainty reporting with cautious domain adaptation.

## 5 Conclusion

While current multimodal LLMs are not suitable for independent patient use, ChatGPT-5 and ChatGPT-4o demonstrated promising performance for psoriasis recognition under the controlled conditions of this single-center image-based study. These findings suggest that such models may have potential as clinician-supervised supportive tools for psoriasis assessment and triage, particularly in settings with limited access to dermatologic expertise. However, broader conclusions regarding clinical utility should be avoided until further validation is performed in external, multi-center, and more heterogeneous real-world datasets.

## Data Availability

The datasets presented in this article are not readily available because due to patient confidentiality and institutional privacy requirements, the underlying clinical photographs and associated patient-level data cannot be publicly shared. De-identified data may still carry a risk of re-identification; therefore, access is strictly restricted in accordance with informed consent and local regulations. Requests to access the datasets should be directed to kiss.norbert@semmelweis.hu.
